# Supplementary material for: Response to Hypersalinity of Four Halophytes Growing in Hydroponic Floating Systems: Prospects in the Phytomanagement of High Saline Wastewaters and Extreme Environments
Source: Plants (Basel). 2023 Apr 22;12(9):1737. doi: 10.3390/plants12091737 (PMC10181242; doi:10.3390/plants12091737)
Supplement: Supplementary file 1 [file plants-12-01737-s001.zip › plants-2315608-supplementary.pdf]

plants

Supplementary Material

Response to Hypersalinity of Four Halophytes Growing in Hydroponic Floating Systems: Prospects in the Phytomanagement of High Saline Wastewaters and Extreme Environments

Meri Barbafieri[1], Francesca Bretzel[1], Andrea Scartazza[1], Daniela Di Baccio[1]*, Irene Rosellini[1], Martina Grifoni[1], Roberto Pini[1], Alice Clementi[2], Elisabetta Franchi[3]*

[1] Research Institute on Terrestrial Ecosystems, National Research Council of Italy (IRET-CNR), Via Moruzzi 1, 56124 Pisa, Italy; meri.barbafieri@cnr.it (M.B.); francesca.bretzel@cnr.it (F.B.); andrea.scartazza@cnr.it (A.S.); irene.rosellini@cnr.it (I.R.); grifonimar@gmail.com (M.G.); roberto-pini@cnr.it (R.P.)

[2] Eni S.p.A., Subsurface and Wells R&D Projects, Via Maritano 26, San Donato Milanese, 20097 Milan, Italy; alice.clementi@eni.com

[3] Eni S.p.A., R&D Environmental &Biological Laboratories, Via Maritano 26, San Donato Milanese, 20097 Milan, Italy

* Correspondence: daniela.dibaccio@cnr.it (D.D.B.); elisabetta.franchi@eni.com (E.F.)

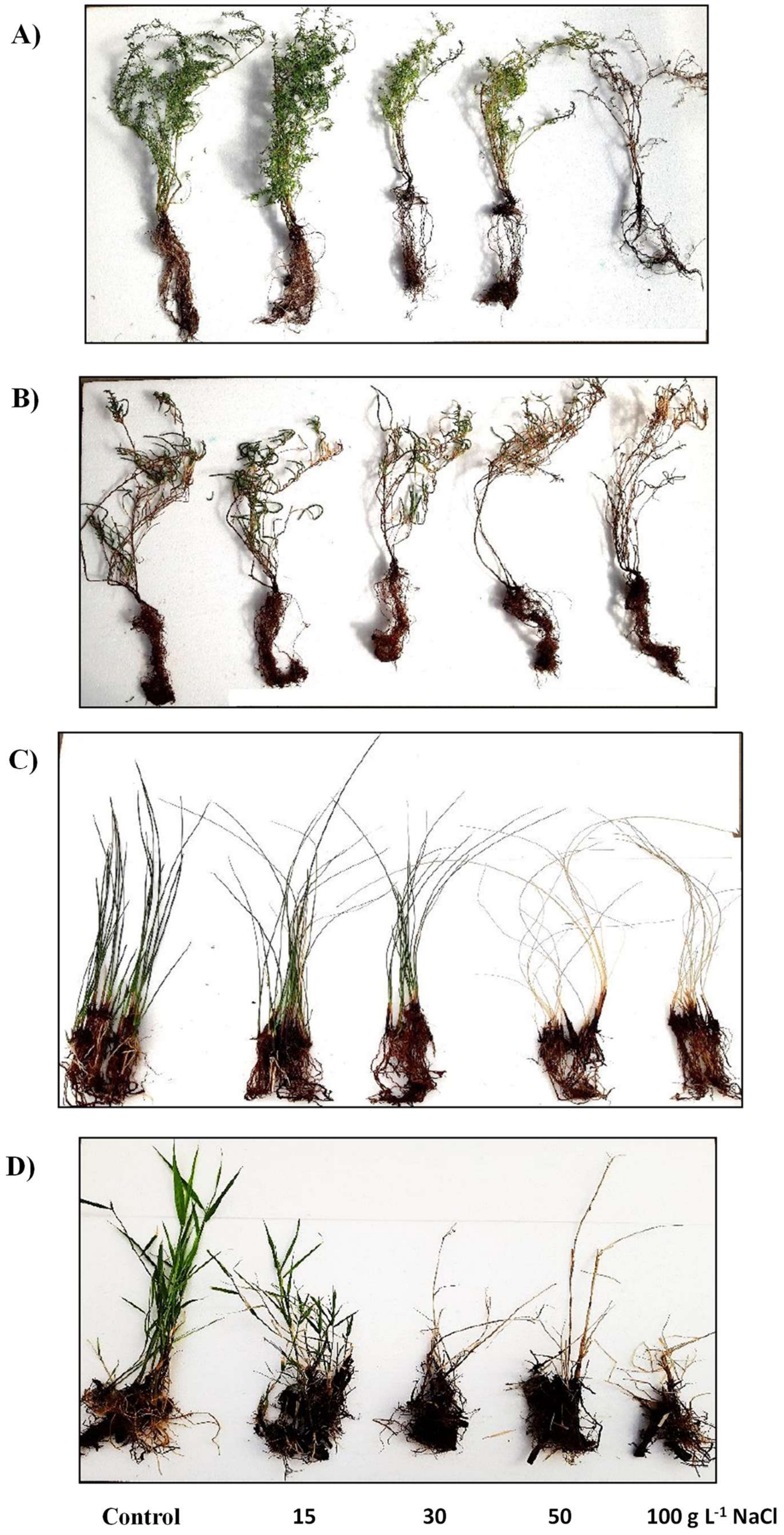

Supplementary Figure S1. *Suaeda fruticosa* (**A**), *Halocnemum strobilaceum* (**B**), *Juncus maritimus* (**C**) and *Phragmites australis* (**D**) after four weeks of treatment with 0 (Control), 15, 30, 50 and 100 g L^{-1} NaCl (corresponding to 0, 257, 514, 856 and 1712 mM NaCl, respectively).

Supplementary Table S1. Fresh weight (FW, g plant^{-1}) of different organs of the four halophyte plant species treated with 0, 15, 30, 50 and 100 g L^{-1} NaCl (corresponding to 0, 257, 514, 856 and 1712 mM NaCl, respectively) for 28 days. Values are means ± standard error (SE) of four plants (n = 4). Results of the two-way ANOVA ($P \leq 0.05$) for the effect of NaCl treatments and species, and of their interaction are shown (F and P values). When the interaction between factors was significant, the Fisher LSD-test ($P \leq 0.05$) was applied: significantly different data are followed by different letters within each column; values followed by a different lower case letter are significantly different across species and NaCl treatments.

		Suaeda fruticosa		
NaCl treatment	leaf FW	stem FW	root FW	total FW
Control (0 g L^{-1})	19.10±2.65 b	9.80±1.60 c-f	5.40±0.40 d	34.30±4.65 cd
15 g L^{-1}	25.60±1.40 a	12.20±2.40 cd	4.41±0.40 d	42.21±2.80 b
30 g L^{-1}	6.80±0.60 f-h	6.65±1.35 c-l	2.81±0.20 d	16.26±1.75 g-l
50 g L^{-1}	8.60±1.20 ef	12.78±3.03 c	5.56±1.51 d	26.93±5.74 d-f
100 g L^{-1}	4.36±0.20 g-i	6.10±0.35 f-l	3.17±0.29 d	13.63±0.84 il
		Halocnemum strobilaceum		
NaCl treatment	leaf FW	stem FW	root FW	total FW
Control (0 g L^{-1})	12.43±1.03 cd	5.80±1.00 g-l	5.40±0.40 d	23.13±2.45 e-g
15 g L^{-1}	9.99±1.56 cd	4.91±0.55 il	3.81±0.83 d	18.70±1.85 e-h
30 g L^{-1}	12.20±0.90 cd	5.20±0.70 h-l	5.25±0.90 d	22.65±2.50 e-g
50 g L^{-1}	10.43±0.83 de	4.67±0.53 il	4.77±1.07 d	19.87±0.77 e-i
100 g L^{-1}	4.28±0.42 g-l	4.25±0.64 l	2.76±0.47 d	11.29±0.25 l
		Juncus maritimus		
NaCl treatment	shoot FW	rhizome FW	roots FW	total FW
Control (0 g L^{-1})	13.73±1.18 c	9.75±0.55 c-g	26.80±1.95 a	50.28±0.22 a
15 g L^{-1}	12.15±1.65 cd	8.93±0.63 c-h	22.85±1.30 b	43.93±2.33 ab
30 g L^{-1}	7.25±0.30 fg	9.60±0.95 c-g	25.83±2.08 ab	42.68±3.33 ab
50 g L^{-1}	4.39±0.29 g-i	8.65±1.05 c-i	11.45±1.70 c	24.49±3.04 ef
100 g L^{-1}	4.12±0.37 h-l	10.25±1.20 c-e	13.90±1.20 c	28.27±2.77 de
		Phragmites australis		
NaCl treatment	shoot FW	rhizome FW	roots FW	total FW
Control (0 g L^{-1})	7.80±1.50 ef	26.58±1.88 b	4.62±0.68 d	39.00±1.06 bc
15 g L^{-1}	4.12±0.80 h-l	31.40±2.25 a	5.23±0.38 d	40.75±3.42 bc
30 g L^{-1}	1.43±0.21 il	11.60±1.20 cd	3.36±0.12 d	16.39±1.53 g-l
50 g L^{-1}	1.79±0.21 il	11.58±1.48 cd	3.35±0.22 d	16.71±1.46 f-l
100 g L^{-1}	1.31±0.12 l	10.35±1.45 c-e	2.81±0.29 d	14.47±1.86 h-l
Statistics		Two-way ANOVA – F (P)		
NaCl	76.64 (<0.001)	19.07 (<0.001)	16.57 (<0.001)	48.25 (<0.001)
Species	78.77 (<0.001)	85.41 (<0.001)	311.02 (<0.001)	43.65 (<0.001)
NaCl x species	12.94 (<0.001)	13.46 (<0.001)	11.67 (<0.001)	6.81 (0.001)

Supplementary Table S2. Water status and relative biomass partitioning between aerial parts and roots of the four halophytic plant species treated with 0 (control), 15, 30, 50 and 100 g L^{-1} NaCl (corresponding to 0, 257, 514, 856 and 1712 mM NaCl, respectively) for four weeks. Values are means ± standard error (SE) of four plants (n = 4). Statistics as in Table S1. RWC (%), relative water content as percentage of fresh weight, SMR, shoot mass ratio, and RMR, root mass ratio, were calculated as in Material and Methods. Before statistical analysis, percentage data were subjected to an arc sine or angular transformation.

	Suaeda fruticosa					
NaCl	leaf RWC	stem RWC	root RWC	root/shoot	SMR	RMR
Control (0 g L^{-1})	80.39±1.31	56.67±1.49	43.33±0.02	0.39±0.02 g-i	0.72±0.01 bc	0.28±0.01 cd
15 g L^{-1}	84.48±0.25	58.07±1.12	46.64±0.75	0.26±0.01 i	0.79±0.005 a	0.21±0.005 e
30 g L^{-1}	79.77±0.90	32.46±16.23	40.56±13.93	0.29±0.05 hi	0.78±0.03 ab	0.22±0.03 de
50 g L^{-1}	83.16±3.57	60.79±13.10	56.68±16.87	0.36±0.01 g-i	0.74±0.01 a-c	0.26±0.01 c-e
100 g L^{-1}	79.10±1.82	57.04±3.04	34.23±3.45	0.60±0.10 f-h	0.63±0.04 d	0.37±0.04 b
	Halocnemum strobilaceum					
NaCl	leaf RWC	stem RWC	root RWC	root/shoot	SMR	RMR
Control (0 g L^{-1})	73.67±1.13	33.43±7.08	40.54±0.96	0.41±0.01 g-i	0.71±0.003 c	0.29±0.003 c
15 g L^{-1}	68.10±6.14	39.94±2.60	34.89±25.31	0.37±0.03 g-i	0.73±0.02 bc	0.27±0.02 cd
30 g L^{-1}	73.85±3.40	30.43±3.98	43.69±4.03	0.43±0.04 g-i	0.70±0.02 c	0.30±0.02 c
50 g L^{-1}	73.46±3.25	29.39±16.96	49.05±3.43	0.41±0.08 g-i	0.71±0.04 bc	0.29±0.04 c
100 g L^{-1}	67.37±1.80	33.09±19.67	50.20±7.88	0.33±0.06 g-i	0.75±0.03 a-c	0.25±0.03 c-e
	Juncus maritimus					
NaCl	shoot RWC	rhizome RWC	root RWC	root/shoot	SMR	RMR
Control (0 g L^{-1})	68.07±1.06	72.88±4.79	82.25±2.25	1.08±0.002 de	0.37±0.01 e	0.40±0.02 ab
15 g L^{-1}	63.62±3.34	64.28±9.22	76.69±0.12	1.22±0.01 cd	0.34±0.01 e	0.42±0.01 ab
30 g L^{-1}	60.87±0.58	67.00±1.84	80.05±0.05	1.81±0.09 a	0.26±0.02 f	0.46±0.004 a
50 g L^{-1}	63.02±1.20	69.91±0.50	78.56±0.87	1.50±0.02 bc	0.24±0.002 f	0.37±0.001 b
100 g L^{-1}	59.78±0.70	70.01±0.10	79.80±0.43	1.70±0.001 ab	0.22±0.001 fg	0.37±0.002 b
	Phragmites australis					
NaCl	shoot RWC	rhizome RWC	root RWC	root/shoot	SMR	RMR
Control (0 g L^{-1})	79.76±1.26	72.35±2.52	87.63±0.58	0.38±0.09 g-i	0.16±0.02 gh	0.06±0.01 g
15 g L^{-1}	67.87±4.86	74.77±1.01	84.60±4.06	0.63±0.18 fg	0.13±0.002 h	0.08±0.02 fg
30 g L^{-1}	69.27±9.82	72.62±0.12	87.58±2.40	1.02±0.25 de	0.11±0.03 h	0.10±0.01 fg
50 g L^{-1}	59.70±0.71	65.21±1.60	81.94±2.38	0.87±0.25 ef	0.13±0.01 h	0.12±0.03 fg
100 g L^{-1}	61.67±9.76	69.44±1.07	80.56±2.57	1.12±0.13 de	0.12±0.02 h	0.13±0.01 f
Statistics			Two-way ANOVA – F (*P*)			
NaCl	2.36 (0.088)	0.69 (0.605)	0.36 (0.833)	9.76 (<0.001)	7.08 (0.001)	2.01 (0.132)
species	20.83 (<0.001)	20.97 (<0.001)	38.10 (<0.001)	117.97 (<0.001)	1044.10 (<0.001)	190.73 (<0.001)
NaCl x species	1.14 (0.382)	0.63 (0.793)	0.57 (0.841)	3.05 (0.013)	4.75 (0.001)	4.81 (0.001)

Supplementary Table S3. Chlorophyll *a* (Chl *a*) and chlorophyll *b* (Chl *b*) in leaves of *S. fruticosa*, *H. strobilaceum*, *J. maritimus* and *P. autralis* exposed for 28 days to increasing NaCl concentrations (0 – Control, 15, 30, 50 and 100 g L^{-1} NaCl, corresponding to 0, 257, 514, 856 and 1712 mM NaCl). Data (mean values ± SE) reported refer to at least three extractions or replications (n = 3-5). For each time point (7, 14, 21 and 28 days of NaCl exposure), the results of a one way-ANOVA (NaCl-treated plants against controls) are indicated (*, $P \leq 0.05$; **, $P \leq 0.01$; ***, $P \leq 0.001$; n. s., not significant, $P > 0.05$). Different letters correspond to significant differences for the Fisher's LSD post-hoc test ($P \leq 0.05$).

Chl *a*	*Suaeda fruticosa*			
Time	7 days	14 days	21 days	28 days
Control (0 g L^{-1})	7.70±0.45 a	3.65±0.75 a	6.63±0.41 a	6.26±0.27 a
15 g L^{-1}	4.60±1.10 b	4.42±0.72 ab	4.12±0.23 b	5.75±0.18 a
30 g L^{-1}	3.98±0.31 b	1.89±0.38 b	1.65±0.11 c	2.63±0.17 b
50 g L^{-1}	4.65±1.23 b	1.55±0.57 b	2.24±0.12 c	2.29±0.04 b
100 g L^{-1}	1.18±0.09 c	1.76±0.61 b	1.52±0.31 c	0.56±0.06 c
one way-ANOVA (*P*)	**	*	***	***
	Halocnemum strobilaceum			
Time	7 days	14 days	21 days	28 days
Control (0 g L^{-1})	3.08±0.09 a	3.15±0.52 a	3.16±0.76 a	3.05±0.05 a
15 g L^{-1}	1.94±0.06 c	2.27±0.36 ab	2.32±0.21 ab	2.88±0.04 a
30 g L^{-1}	2.39±0.15 b	1.89±0.08 ab	2.04±0.22 b	3.22±0.27 a
50 g L^{-1}	1.65±0.08 cd	1.48±0.08 b	2.31±0.11 b	2.16±0.11 b
100 g L^{-1}	1.39±0.04 d	1.70±0.36 b	1.19±0.11 c	0.71±0.03 c
one way-ANOVA (*P*)	***	0.089	**	***
	Juncus maritimus			
Time	7 days	14 days	21 days	28 days
Control (0 g L^{-1})	2.77±0.31 a	2.10±0.06 b	2.55±0.26 a	4.66±0.41 a
15 g L^{-1}	3.03±0.20 a	0.42±0.03 d	1.19±0.07 b	1.72±0.07 b
30 g L^{-1}	2.91±0.10 a	2.56±0.21 a	0.94±0.05 b	1.76±0.06 b
50 g L^{-1}	2.14±0.06 b	0.98±0.01 c	0.46±0.08 c	0.17±0.01 c
100 g L^{-1}	2.19±0.06 b	0.70±0.06 cd	0.56±0.02 c	0.49±0.03 c
one way-ANOVA (*P*)	**	***	***	***
	Phragmites australis			
Time	7 days	14 days	21 days	28 days
Control (0 g L^{-1})	2.98±0.97	3.60±1.34	6.79±1.39	5.19±0.30 a
15 g L^{-1}	1.51±0.07	2.52±0.76	7.87±2.48	4.37±0.28 b
30 g L^{-1}	1.22±0.40	0.74±0.21		
50 g L^{-1}	2.19±0.47	1.72±0.27		
100 g L^{-1}	1.23±0.27	1.04±0.30		
one way-ANOVA (*P*)	n. s.	n. s.	n. s.	*

Supplementary Table S4. Chlorophyll *b* (Chl *b*) in leaves of *S. fruticosa*, *H. strobilaceum*, *J. maritimus* and *P. autralis* (D) exposed for 28 days to increasing NaCl concentrations (0, Control; 15, 30, 50, and 100 g L^{-1} NaCl, corresponding to 0, 257, 514, 856 and 1712 mM NaCl). Data (mean values ± SE) reported refer to at least three extractions or replications (*n* = 3-5). Statistics as in Table S3.

Chl *b*		*Suaeda fruticosa*		
Time	7 days	14 days	21 days	28 days
Control (0 g L^{-1})	3.00±0.32 a	1.42±0.21 ab	2.36±0.15 a	2.13±0.16 a
15 g L^{-1}	1.84±0.38 b	1.61±0.25 a	1.45±0.08 b	1.97±0.14 a
30 g L^{-1}	1.61±0.04 b	0.76±0.15 b	0.66±0.04 c	0.91±0.09 b
50 g L^{-1}	1.95±0.47 b	0.71±0.26 b	0.91±0.06 c	0.89±0.02 b
100 g L^{-1}	0.45±0.06 c	0.83±0.29 b	0.84±0.16 c	0.35±0.03 b
one way-ANOVA (*P*)	**	n. s. (0.072)	***	***
		Halocnemum strobilaceum		
Time	7 days	14 days	21 days	28 days
Control (0 g L^{-1})	1.11±0.03 a	0.96±0.05 a	1.11±0.25 a	1.02±0.02 a
15 g L^{-1}	0.70±0.01 c	0.70±0.003 ab	0.82±0.08 b	1.00±0.01 a
30 g L^{-1}	0.86±0.06 b	0.61±0.11 b	0.78±0.06 b	1.10±0.11 a
50 g L^{-1}	0.64±0.06 cd	0.47±0.05 b	0.83±0.03 b	0.78±0.01 b
100 g L^{-1}	0.51±0.02 d	0.59±0.09 b	0.48±0.03 c	0.34±0.01 c
one way-ANOVA (*P*)	**	*	**	***
		Juncus maritimus		
Time	7 days	14 days	21 days	28 days
Control (0 g L^{-1})	0.93±0.10 a	0.66±0.02 b	0.82±0.09 a	1.46±0.12 a
15 g L^{-1}	0.96±0.05 a	0.16±0.01 e	0.43±0.02 b	0.55±0.02 b
30 g L^{-1}	0.96±0.01 a	0.80±0.06 a	0.31±0.02 b	0.56±0.02 b
50 g L^{-1}	0.75±0.01 b	0.48±0.04 c	0.28±0.05 b	0.11±0.01 c
100 g L^{-1}	0.73±0.01 b	0.30±0.03 d	0.39±0.05 b	0.33±0.02 d
one way-ANOVA (*P*)	**	***	***	***
		Phragmites australis		
Time	7 days	14 days	21 days	28 days
Control (0 g L^{-1})	0.89±0.97	1.08±0.42	2.11±0.43	1.52±0.10 a
15 g L^{-1}	0.41±0.07	0.67±0.19	2.09±0.63	1.28±0.11 b
30 g L^{-1}	0.45±0.40	0.28±0.09		
50 g L^{-1}	0.79±0.47	1.73±0.16		
100 g L^{-1}	0.40±0.27	0.38±0.11		
one way-ANOVA (*P*)	n. s.	n. s.	n. s.	*

Supplementary Table S5. Nitrogen to phosphorous ratio (N:P) in different organs of the four halophyte plant species treated with 0, 15, 30, 50 and 100 g L^{-1} NaCl (corresponding to 0, 257, 514, 856 and 1712 mM NaCl, respectively) for 28 days. Two-way ANOVA. Values are means ± standard deviation (SD) of two-three extractions (n = 2-3) derived from bulked vegetal material of four plants for each species and NaCl treatment. Statistics as in Table S1.

	Suaeda fruticosa		
NaCl treatment	leaf N:P	stem N:P	root N:P
Control (0 g L^{-1})	15.56±1.56 f	11.95±0.75 e-g	8.47±0.55 c
15 g L^{-1}	20.77±0.79 de	11.08±0.02 e-h	5.68±0.59 c
30 g L^{-1}	22.27±1.93 cd	13.36±2.07 d-f	6.21±0.77 c
50 g L^{-1}	18.87±0.97 e	7.09±0.93 i-n	2.99±0.34 c
100 g L^{-1}	10.44±0.27 gh	5.49±0.12 mn	3.97±0.24 c
	Halocnemum strobilaceum		
NaCl treatment	leaf N:P	stem N:P	root N:P
Control (0 g L^{-1})	22.62±0.46 cd	16.48±1.24 bc	13.73±0.73 c
15 g L^{-1}	23.95±1.26 c	15.60±0.94 cd	12.91±0.08 c
30 g L^{-1}	26.79±1.38 b	18.90±4.04 b	12.95±0.19 c
50 g L^{-1}	31.22±3.73 a	14.04±3.18 c-e	14.21±1.28 c
100 g L^{-1}	15.53±0.82 f	6.94±0.31 l-n	8.80±0.04 c
	Juncus maritimus		
NaCl treatment	shoot N:P	rhizome N:P	roots N:P
Control (0 g L^{-1})	14.85±2.03 f	22.36±1.37 a	397.30±139.06 a
15 g L^{-1}	19.28±0.18 e	11.46±2.09 e-h	207.35±71.54 b
30 g L^{-1}	8.09±0.30 hi	5.60±0.79 mn	n. d.
50 g L^{-1}	11.58±0.59 g	5.35±0.96 n	48.28±9.72 c
100 g L^{-1}	7.33±0.14 i	4.10±0.22 n	48.61±12.31 c
	Phragmites australis		
NaCl treatment	shoot N:P	rhizome N:P	roots N:P
Control (0 g L^{-1})	10.38±0.30 gh	10.09±0.28 g-i	14.40±5.04 c
15 g L^{-1}	7.88±0.29 hi	11.45±0.38 e-h	9.80±2.45 c
30 g L^{-1}	7.07±0.25 hi	8.49±0.64 h-m	5.72±1.31 c
50 g L^{-1}	7.58±0.35 i	9.99±1.09 g-l	7,23±2.39 c
100 g L^{-1}	7.79±0.41 hi	10.85±0.30 f-h	12.76±4.72 c
Statistics	Two-way ANOVA – F (*P*)		
NaCl	36.47 (<0.001)	36.84 (<0.001)	6.86 (0.003)
Species	242.16 (<0.001)	22.74 (<0.001)	24.26 (<0.001)
NaCl x species	15.77 (<0.001)	14.91 (<0.001)	6.10 (0.001)

Supplementary Table S6. Correlations coefficients (R2) between growth, physiological and chemical attributes in leaves or shoot of *S. fruticosa* (A), *H. strobilaceum* (B), *J. maritimus* (C), and *P. australis* (D) after 28 days of treatment with 0, 15, 30, 50 and 100 g L^{-1} NaCl (corresponding to 0, 257, 514, 856 and 1712 mM NaCl, respectively). Combinations which performed R2 ≥ 0.570 are indicated in bold.

A) *Suaeda fruticosa*

	leaf DW	leaf RWC	Fv/Fm	Φ PSII	NPQ	Chl *a*	Chl *b*	Car	Chl *a*+*b*	Chl *a*/*b*	N	P	K	Ca	Mg	Na	K/Na	Ca/Na
leaf DW	1.00																	
leaf RWC	0.37	1.00																
Fv/Fm	**0.58**	0.41	1.00															
Φ PSII	**0.62**	0.46	**0.93**	1.00														
NPQ	-0.11	0.08	0.54	0.23	1.00													
Chl *a*	**0.96**	0.33	**0.75**	**0.79**	0.02	1.00												
Chl *b*	**0.96**	0.34	**0.74**	**0.78**	0.04	**0.99**	1.00											
Car	**0.89**	0.37	**0.86**	**0.86**	0.23	**0.97**	**0.98**	1.00										
Chl *a* + *b*	**0.96**	0.33	**0.75**	**0.79**	0.02	**0.99**	**0.99**	**0.97**	1.00									
Chl *a*/*b*	**0.69**	0.33	**0.96**	**0.94**	0.37	**0.85**	**0.83**	**0.91**	**0.84**	1.00								
N	**0.72**	0.04	0.18	0.29	-0.38	**0.70**	**0.68**	0.56	**0.69**	0.36	1.00							
P	0.01	-0.36	**-0.68**	**-0.59**	**-0.64**	-0.14	-0.13	-0.30	-0.13	**-0.57**	0.54	1.00						
K	-0.30	0.17	-0.40	-0.39	-0.20	-0.46	-0.46	-0.49	-0.46	-0.42	**-0.58**	-0.22	1.00					
Ca	-0.40	-0.52	**-0.81**	**-0.78**	-0.41	-0.49	-0.49	**-0.60**	-0.49	**-0.76**	0.20	**0.89**	-0.19	1.00				
Mg	0.18	-0.34	-0.16	-0.11	-0.26	0.20	0.21	0.12	0.21	-0.08	**0.69**	**0.76**	**-0.80**	**0.67**	1.00			
Na	**0.59**	0.34	**0.97**	**0.94**	0.45	**0.76**	**0.75**	**0.85**	**0.76**	**0.97**	0.18	-0.70	-0.32	**-0.85**	-0.23	1.00		
K/Na	-0.54	-0.18	**-0.90**	**-0.84**	-0.49	**-0.75**	**-0.74**	**-0.84**	**-0.75**	**-0.89**	-0.40	0.40	**0.76**	0.49	-0.25	**-0.85**	1.00	
Ca/Na	-0.53	-0.45	**-0.98**	**-0.92**	-0.54	**-0.69**	**-0.68**	**-0.81**	**-0.68**	**-0.94**	-0.05	**0.79**	0.23	**0.91**	0.34	**-0.98**	**0.81**	1.00

B) *Halocnemum strobilaceum*

	leaf DW	leaf RWC	Fv/Fm	Φ PSII	NPQ	Chl *a*	Chl *b*	Car	Chl *a*+*b*	Chl *a*/*b*	N	P	K	Ca	Mg	Na	K/Na	Ca/Na
leaf DW	1.00																	
leaf RWC	0.28	1.00																
Fv/Fm	**0.95**	0.42	1.00															
Φ PSII	**0.94**	0.18	**0.88**	1.00														
NPQ	0.10	0.38	0.24	-0.16	1.00													
Chl *a*	**0.91**	0.46	**0.90**	**0.93**	0.03	1.00												
Chl *b*	**0.89**	0.45	**0.88**	**0.91**	0.00	**0.99**	1.00											
Car	**0.89**	0.49	**0.89**	**0.88**	0.08	**0.99**	**0.99**	1.00										
Chl *a*+*b*	**0.91**	0.45	**0.90**	**0.92**	0.02	**0.99**	**0.99**	**0.99**	1.00									
Chl *a*/*b*	**0.96**	0.43	**0.94**	**0.88**	0.29	**0.91**	**0.87**	**0.89**	**0.90**	1.00								
N	0.21	0.21	0.12	0.19	-0.20	0.10	0.06	0.03	0.09	0.20	1.00							
P	**-0.63**	-0.32	**-0.71**	-0.52	-0.49	**-0.60**	**-0.60**	**-0.65**	**-0.60**	**-0.68**	0.43	1.00						
K	**0.72**	0.49	**0.80**	0.55	**0.59**	**0.60**	**0.57**	**0.62**	**0.60**	**0.80**	0.17	**-0.77**	1.00					
Ca	**-0.86**	-0.47	**-0.82**	**-0.78**	-0.14	**-0.85**	**-0.82**	**-0.84**	**-0.85**	**-0.87**	-0.48	0.39	**-0.66**	1.00				
Mg	**-0.92**	-0.44	**-0.91**	**-0.82**	-0.28	**-0.93**	**-0.91**	**-0.95**	**-0.92**	**-0.93**	0.04	**0.80**	**-0.76**	**0.82**	1.00			
Na	**0.76**	0.18	**0.75**	**0.81**	-0.06	**0.78**	**0.78**	**0.78**	**0.78**	**0.74**	-0.29	**-0.75**	0.43	-0.47	**-0.78**	1.00		
K/Na	**0.70**	0.49	**0.78**	0.53	**0.60**	**0.58**	0.55	**0.59**	**0.58**	**0.79**	0.19	**-0.75**	**0.99**	**-0.66**	**-0.74**	0.39	1.00	
Ca/Na	**-0.92**	-0.45	**-0.88**	**-0.86**	-0.12	**-0.92**	**-0.89**	**-0.91**	**-0.91**	**-0.92**	-0.37	0.50	**-0.69**	**0.98**	**0.89**	**-0.62**	**-0.68**	1.00

C) *Juncus maritimus*

	leaf DW	leaf RWC	Fv/Fm	Φ PSII	NPQ	Chl *a*	Chl *b*	Car	Chl *a*+*b*	Chl *a*/*b*	N	P	K	Ca	Mg	Na	K/Na	Ca/Na
leaf DW	1.00																	
leaf RWC	**0.64**	1.00																
Fv/Fm	**0.89**	0.42	1.00															
Φ PSII	**0.89**	0.44	**0.99**	1.00														
NPQ	**0.80**	0.34	**0.90**	**0.88**	1.00													
Chl *a*	**0.78**	**0.69**	**0.73**	**0.77**	0.53	1.00												
Chl *b*	**0.74**	**0.66**	**0.67**	**0.71**	0.47	**0.99**	1.00											
Car	**0.78**	**0.66**	**0.78**	**0.81**	**0.58**	**0.99**	**0.98**	1.00										
Chl *a*+*b*	**0.77**	**0.69**	**0.72**	**0.75**	0.51	**0.99**	**0.99**	**0.99**	1.00									
Chl *a*/*b*	**0.89**	0.44	**0.99**	**0.99**	**0.90**	**0.75**	**0.69**	**0.79**	**0.74**	1.00								
N	0.46	**0.80**	0.10	0.11	0.01	0.41	0.39	0.32	0.41	0.12	1.00							
P	**-0.80**	**-0.60**	**-0.62**	**-0.61**	**-0.59**	-0.43	-0.35	-0.39	-0.42	**-0.62**	**-0.68**	1.00						
K	**-0.96**	**-0.70**	**-0.76**	**-0.76**	**-0.61**	**-0.82**	**-0.79**	**-0.78**	**-0.81**	**-0.75**	**-0.64**	**0.79**	1.00					
Ca	**-0.70**	-0.53	**-0.85**	**-0.87**	**-0.74**	**-0.76**	**-0.68**	**-0.80**	**-0.74**	**-0.87**	-0.18	0.54	**0.59**	1.00				
Mg	-0.40	-0.11	-0.16	-0.17	-0.07	-0.42	-0.49	-0.37	-0.44	-0.16	-0.23	0.05	0.52	-0.15	1.00			
Na	**-0.65**	-0.18	**-0.65**	**-0.65**	**-0.57**	**-0.66**	**-0.69**	**-0.67**	**-0.67**	**-0.63**	0.08	0.11	**0.61**	0.32	**0.71**	1.00		
K/Na	**0.65**	**0.58**	0.53	0.56	0.36	**0.92**	**0.95**	**0.89**	**0.93**	0.54	0.35	-0.21	**-0.73**	-0.48	**-0.65**	**-0.78**	1.00	
Ca/Na	**0.65**	**0.58**	0.49	0.51	0.31	**0.88**	**0.93**	**0.85**	**0.89**	0.49	0.41	-0.24	**-0.76**	-0.39	**-0.72**	**-0.78**	**0.99**	1.00

D) *Phragmites australis*

	leaf DW	leaf RWC	Fv/Fm	Φ PSII	NPQ	Chl *a*	Chl *b*	Car	Chl *a*+*b*	Chl *a*/*b*	N	P	K	Ca	Mg	Na	K/Na	Ca/Na
leaf DW	1.00																	
leaf RWC	**0.95**	1.00																
Fv/Fm	**0.93**	**0.94**	1.00															
Φ PSII	**0.96**	**0.94**	**0.99**	1.00														
NPQ	**0.90**	**0.91**	**0.99**	**0.99**	1.00													
Chl *a*	**0.96**	**0.97**	**0.99**	**0.98**	**0.96**	1.00												
Chl *b*	**0.96**	**0.97**	**0.98**	**0.98**	**0.96**	**0.99**	1.00											
Car	**0.98**	**0.97**	**0.98**	**0.98**	**0.95**	**0.98**	**0.97**	1.00										
Chl *a*+*b*	**0.96**	**0.97**	**0.99**	**0.98**	**0.96**	**0.99**	**0.99**	**0.96**	1.00									
Chl *a*/*b*	**0.93**	**0.94**	**0.99**	**0.99**	**0.99**	**0.99**	**0.98**	**0.97**	**0.98**	1.00								
N	**0.86**	**0.83**	**0.85**	**0.84**	**0.80**	**0.91**	**0.91**	**0.91**	**0.91**	**0.85**	1.00							
P	**0.59**	0.42	**0.67**	**0.67**	**0.68**	**0.63**	**0.63**	**0.63**	**0.63**	**0.67**	**0.70**	1.00						
K	**0.90**	**0.97**	**0.95**	**0.95**	**0.94**	**0.94**	**0.93**	**0.93**	**0.94**	**0.95**	**0.71**	0.41	1.00					
Ca	**0.83**	**0.76**	**0.93**	**0.92**	**0.93**	**0.89**	**0.89**	**0.88**	**0.89**	**0.93**	**0.83**	**0.89**	**0.77**	1.00				
Mg	**0.84**	**0.96**	**0.86**	**0.86**	**0.84**	**0.88**	**0.88**	**0.88**	**0.88**	**0.86**	**0.67**	0.21	**0.97**	**0.62**	1.00			
Na	**-0.57**	**-0.75**	-0.55	-0.55	-0.53	**-0.57**	**-0.57**	**-0.57**	**-0.57**	-0.55	-0.30	0.24	**-0.78**	-0.21	**-0.89**	1.00		
K/Na	**0.88**	**0.94**	**0.83**	**0.83**	**0.77**	**0.91**	**0.91**	**0.91**	**0.91**	**0.83**	**0.90**	0.36	**0.84**	**0.66**	**0.88**	**-0.66**	1.00	
Ca/Na	**0.85**	**0.92**	**0.80**	**0.79**	**0.73**	**0.88**	**0.88**	**0.89**	**0.88**	**0.80**	**0.87**	0.30	**0.82**	**0.61**	**0.88**	**-0.69**	**0.99**	1.00
